# CCRDB: a cancer circRNAs-related database and its application in hepatocellular carcinoma-related circRNAs

**DOI:** 10.1093/database/baz063

**Published:** 2019-06-19

**Authors:** Qingyu Liu, Yanning Cai, Haiquan Xiong, Yiyun Deng, Xianhua Dai

**Affiliations:** 1 School of Electronics and Information Technology, Sun Yat-Sen University, Guangzhou 510006, China; 2 Jinan University, No. 601, West Huangpu Avenue, Guangzhou, Guangdong, China

## Abstract

Circular RNAs (circRNAs) are widely expressed in human cells and tissues and can form a covalently closed exon circularization, which have stable patterns and play important regulatory roles in physiological or pathological process. There is still lack of a comprehensively disease-related knowledge base for in-depth analysis of circRNAs. In this paper, a cancer circRNAs-related database (CCRDB) was established. The CCRDB’s initial circRNAs data were collected by sequencing experimental data of 10 samples from 5 patients with hepatocellular carcinoma (HCC), where a total of 11 501 circRNAs were found and can easily be expanded by collecting and analyzing external data sources such as circBASE (1). Using CCRDB, we have further studied the relationships between circRNAs and HCC and found that circRNAs (hsa_circ_ 0002130, hsa_circ_0084615, hsa_circ_0001445, hsa_circ_0001727 and hsa_circ_0001361) and the corresponding genes ID [C3 (2, 3), ASPH (4), SMARCA5 (5), ZKSCAN1 (6) and FNDC3B (7)], respectively, might be the potential biomarker targets for HCC. Furthermore, our experiment also found that some new circRNAs chromosome sites chr12:23998917 24048958 and chr16:72090429 72093087 and the corresponding genes ID (SOX5 (8) and HP (9), respectively), might be the potential biomarker targets for HCC. These results indicate that CCRDB can effectively reveal the relationships between circRNAs and HCC. As the first circRNAs database to provide analysis and comparison functions, it is of great significance for researchers to further study the rules of circRNAs, to understand the causes of circRNAs in disease discovery and to find target genes for therapeutic approaches.

## Introduction

Circular RNA (or circRNAs) is a type of noncoding RNA that forms a covalently closed continuous loop with the 3′ and 5′ ends binding together. This feature confers numerous properties to circRNAs, many of which have only been identified recently. Some circRNAs can act as microRNA sponges to block the function of microRNAs, thereby affecting gene regulation and expression, and are widely involved in life activities and play important regulatory roles in tumorigenesis and development (10–11). For example, circRNAs CDR1as/ciRS-7 (a circular RNA sponge for antisense of microRNAs-7 or CDR1) inhibits the expression of microRNAs-7, thereby increasing expression of the target gene of microRNAs-7. Sex-determining region on Y chromosome gene (Sry) has also been shown to be used as a sponge of microRNAs-138 (12). Liu *et al*. found that circRNAs-CER regulates MMP13 expression by acting as a competitive endogenous RNA (ceRNA) (13). Other studies have shown that circRNA is involved in the development of various diseases, including atherosclerosis, neurological diseases and cancer (14–15). Guarnerio *et al*. (16) found that tumors carrying chromosomal translocations also contained circRNAs from rearranged genomes: abnormal fusion of circRNA (f-circRNA). They further confirmed that these circRNAs may be functionally relevant in promoting tumorigenesis, suggesting their diagnostic and therapeutic potential. Meanwhile, the development of high-throughput sequencing technology (17) has greatly expanded the scope of transcriptome research and provided a way to view circRNAs in different samples. However, the specific role of most circRNAs has not yet been identified.

At present, hepatocellular carcinoma (HCC) is one of the most common malignancies and the sixth largest cancer killer in the world. Most HCC is caused by chronic hepatitis B virus infection and subsequent cirrhosis (18). It has been reported that the fact that a cellular circRNA has been found stable in saliva (19) and exosome (20) makes circRNA a promising biomarker for diagnosis. Similarly, some studies have shown that if the expression of microarray-7(miR-7) is up-regulated in HCC cells, the cell cycle may be stagnated in G1/S phase, thus inhibiting the proliferation of cancer cells (21). In recent years, Qin *et al*. found that hsa-circ_0005075 is a potential target for diagnosis and treatment of HCC. Their results showed that circRNA can successfully distinguish between tumors and normal samples (22, 23). Li *et al*. found that CIRCMTO1 could act as a sponge of carcinogenic microRNA9 to up-regulate the expression of p21 and significantly affect the proliferation of HCC cells. CIRCMTO1 might be used as a prognostic factor and therapeutic target for HCC (24). Huang *et al*. found that has_circrna_100338 inhibited the expression of microRNA141-3P and played an important role in the regulation of metastatic potential of HCC cells and provided one of the first circRNAs biomarkers for HCC clinical studies (25). Fu *et al*. showed that HSA-CIRC_0353570 was closely related to the clinicopathological characteristics of HCC patients. The background of liver cirrhosis was related to the decrease of HSA-CIRC_0353570 (26). Chen found that HSAXCIRCY05996 interacted with microRNAs-129-5P and regulated Notch1 mRNA expression by acting as a sponge of microRNAs-129-5P. It has been reported that Notch plays an important role in the occurrence and metastasis of HCC (27–29).

In recent years, researchers have paid more and more attention to the study of circRNAs, and many circRNAs-related databases have been published, such as circBase, circNet and database for cancer-specific circRNAs(CSCD) (1, 30–34). Among them, circBase merges and unifies circRNAs data sets from public references and provides evidence to support its expression in the genomic context (29). CircNet provides a common database of tissue-specific circRNAs expression profiles and circRNA–miRNA gene regulatory networks and provides new methods and nomenclature to identify new circRNAs. None of them is specifically targeted at the comparison of disease-related RNAs, and it is difficult to study the biological effects and regulatory mechanisms of disease-related information. CSCD is a comprehensive database of cancer-specific circRNAs that provides general information and regulatory property queries, but it does not provide new RNA discoveries, nor does it provide tools and methods for in-depth analysis.

## Database sources

The CCRDB data sources include our experimental data and external data from other author’s literatures. Several individuals were selected to conduct the experiment. Five pairs of circRNAs differentially expressed in HCC cells and normal tissues adjacent to the cancer were screened. We divided them into two groups: group B was normal cells and group C was HCC cells.

Combining with the published circRNAs database circBASE, we annotated the circRNAs in the samples according to the source region. In our experiment, a total of 11 501 circRNAs were found and listed in Table 1. Compared with the circBASE database, 4989 circRNAs were not included in the circBASE, and they were new circRNAs found in our experiment. Among them, we found 5033, 2446, 3101, 1068 and 2249 circRNAs in normal cells (group B) and 3741, 3233, 2561, 2555 and 2209 circRNAs in cancer cells (group C).

**Table. 1:** Summary of HCC circRNAs

**Samples**	**1B**	**1C**	**2B**	**2C**	**3B**	**3C**	**4B**	**4C**	**5B**	**5C**
Number of circular junction reads	34 101	20 772	12 438	18 293	16 155	12 256	4614	13 300	11 332	9780
Number of circRNA species	5033	3741	2446	3433	3101	2561	1068	2555	2249	2209
Number of circRNA species reported in circBase	3275 (65.07%)	2585 (69.10%)	1767 (72.24%)	2358 (68.69%)	2196 (70.82%)	1847 (72.12%)	747 (69.94%)	1766 (69.12%)	1599 (71.1%)	1682 (76.14%)
Number of circRNA species originated from exon regions	4572 (90.84%)	3421 (91.45%)	2277 (93.09%)	3163 (92.14%)	2848 (91.84%)	2324 (90.75%)	968 (90.64%)	2276 (89.08%)	2062 (91.69%)	2051 (92.85%)
Number of circRNA species originated from intron regions	454 (9.02%)	307 (8.21%)	158 (6.46%)	261 (7.60%)	248 (8.00%)	229 (8.94%)	94 (8.80%)	271 (10.61%)	179 (7.96%)	146 (6.61%)
Number of circRNA species originated from intergenic regions	7 (0.14%)	13 (0.35%)	11 (0.45%)	9 (0.26%)	5 (0.16%)	8 (0.31%)	6 (0.56%)	8 (0.31%)	8 (0.36%)	12 (0.54%)

The CCRDB also collects external data sets from existing circBASE database where thousands of circRNAs have recently been shown to be expressed in *Homo sapiens* cells, which are published from literatures (35–42). This data set consists of basic circRNAs information along with their genomic coordinates, annotation, predicted miRNA seed matches and sample’s junction reads. Other external data is very easily added to the CCRDB database. In total, the CCRDB includes 364 582 circRNAs from 62 human organ samples. Table 2 below shows statistics of the CCRDB.

**Table 2:** Statistics of the CCRDB

**circRNA study**	**# Sample**	**# circRNAs**
Our experiment	10	11 501
Maass 2017	24	8757
Rybak-Wolf 2015	7	165 173
Zhang 2013	1	103
Jeck 2013	1	7771
Salzman 2013	15	168 790
Memczak 2013	4	2487
Total	62	364 582

## Database structure

In CCRDB, we mainly consider three aspects, i.e. circRNAs information, annotation information and analysis information. Major information in the CCRDB is listed in the Table **3** below.

**Table 3:** Major information in CCRDB

**1, CircRNAs information**	
**Field name**	**Description**
Sample type	Sample type, disease name or organ name
Sample_ID	Sample Identifier
CircRNA_ID	CircRNAs Identifier
CircBase_ID	CircBase database Identifier
Chr	Chromosomal localization of circRNAs detected
CircRNA_star	Localization of circRNAs detected at the start site
CircRNA_end	Localization of circRNAs detected at the end side
#Junction_reads	The junction reads number of the circRNAs that support head to tail connection
SM_MS_SMS	CircRNAs reads alignment signal
#non_junction_reads	The number of reads to circRNA that support head to tail flank area (flanking).
Junction_reads_ratio	a parameter that can be used to measure the reliability of circRNAs
CircRNA_type	the circRNA type characterized by the region
Gene_ID	the corresponding gene ID according to the location of circRNAs
**2, CircRNAs differential expression analysis results**	
**Field name**	**Description**
Group ID	A comparison group Identifier of sample B and C
CircRNA_ID	CircRNAs Identifier
CircBase_ID	CircBase database Identifier
Gene ID	The corresponding gene ID according to the location of circRNAs
B-Expression	The number of junction reads that supports the circRNAs head to tail connection in the sample B
C-Expression	The number of junction reads that supports the circRNAs head to tail connection in the C sample
B-TPM	Normalized treatment (TPM) of sample B (When the corresponding circRNAs is not detected in a certain sample, the value will be reset to 0.001.)
C-TPM	Normalized treatment (TPM) of sample C (When the corresponding circRNAs is not detected in a certain sample, the value will be reset to 0.001.)
Log2 Ratio (1C/1B)	Samples B and C’s junction reads that were compared with log2
Up-Down-Regulation	Up or down regulation according to the normalized expression comparison from sample B to C
*P*-value	*P*-value
FDR	FDR for the *P*-value

## Database construction

**Figure 1:**
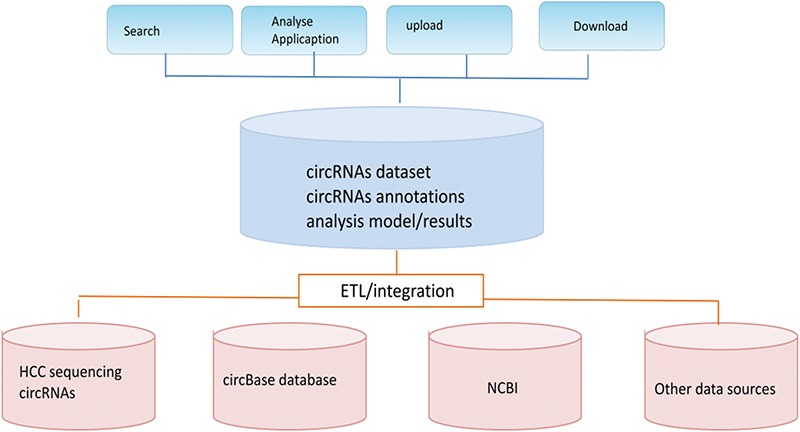
The CCRDB system architecture.

The main purpose of our CCRDB database is to integrate and maintain a high quality circRNAs database and analysis platform to further discover the relationships between circRNAs and HCC. It is a comprehensive and fully functional circRNAs resource library. Figure 1 below illustrates the main structure of the CCRDB, which is based on the client/server architecture. The CCRDB database contains a list of circRNAs, functional annotations and analysis function of the circRNAs.

In terms of data structure, it is implemented by a relational database and a textual database, which can adapt to heterogeneous data. The database implements functions such as data modeling, data extraction, conversion and loading, etc. In order to eliminate differences between data samples from various sources, we label the data according to circRNAs ID and gene ID, which facilitate the implementation of subsequent analysis applications.

## Usage

As a comprehensive and interactive database, CCRDB provides the following main functions, including search, analyse application, download and upload.

**Figure 2:**
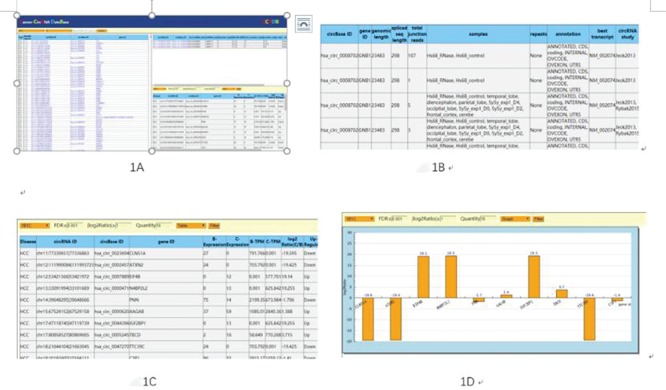
The usage of CCRDB.

Users can browse circRNAs by selecting the sample name, circRNA_ID (for example, Chr X: 891303|892653 representing the donor and receptor sites of each circRNA), circBASE_ID, gene_id and more to get more intuitive information (Figure 2A). All the information will include sample type, circRNA ID, circBase ID, gene ID, sample source and etc. By clicking on any circRNA ID, the circRNA-related chromosome location, start and end sites will be displayed in the upper right corner of the home page. It supports the number of junction reads that are connected at the beginning and the end of circRNAs and supports the aligning of circRNAs. The number of reads aligned to the flanking regions at the ends of the circRNAs is used as a parameter to measure the reliability of circRNAs, junction_reads_ratio and the type of circRNAs in detail (Figure 2B).

We innovatively provide a comparative analysis platform to provide data analysis functions by importing different samples of circRNAs data from different organs. The comparison of two groups of circRNAs data can come from different sources, which is flexible and suitable for various comparative analyses.

The number of junction reads that supports the connection between the head and tail of the circRNAs is used as a comparison criterion to measure the strength of circRNAs signal. The corresponding circRNAs in the sample selected by the user will get the relevant tabular data or up-and-down column analysis diagram (Figure 2C and D) under the selection of samples, FDR and | log2Ratio | numerical settings, number display selection, display mode (table or diagram) and other screening conditions.

We can use the upload function to import data to be analyzed, and its semaphore is based on junction reads. In analysis application, select the circRNAs data of the comparison group to be compared to carry on a pairwise comparison by choosing the result condition (FDR and log2Ratio) (Figure 2B). You can get the differences in the selected comparison group. The result of circRNAs comparison can be expressed by table or graph (Figure 2C and D) for further analytical studies. After selecting several comparison groups for comparison, we can integrate the conclusions of the above comparison groups to get more interesting results. Through comparative analysis, we can obtain the common differencing results from many sample’s circRNAs, such as circRNA signal, intensity, regulatory direction and can distinguish the differences of all circRNAs or parts of different samples, including the number, regulatory direction and semaphore characteristics.

**Table 4:** Comparison of some circRNA databases

	**CircBase**	**CircNEet**	**CSCD**	**CCRDB**
Purpose of the study	An integrated circRNAs database of data from the literature.	A public database that provides tissue-specific circRNAs expression profiles and circRNAs–miRNA gene regulatory networks.	A comprehensive cancer-specific circRNAs database	A circRNAs integration database and tools for analysis function.
Reference source	CircRNAs in scientific literature.	circRNAs in scientific literature.	CircBASE, circNET and other databases.	Experimental sequencing data and related circRNAs literature data.
Analysis function	No	No	No	Yes
Discovery of new CircRNAs	No	Yes	No	Yes
Innovation point	Integrate several circRNAs data into a standardized database.	CircRNAs is classified by new expression pattern, and new circRNAs is found and named.	Provide the first comprehensive cancer-specific circRNAs database.	Provide new circRNAs discovery and analysis tools to search for candidate target genes.

**Table 5:** Statistics of the different circRNAs number

Comparison group	Diff number	Sign. Diff number	Percentiles (%)
1B & 1C	6808	111	1.63
2B & 2C	4652	44	0.95
3B & 3C	4365	21	0.48
4B & 4C	3102	47	1.52
5B & 5C	3534	25	0.71

The Diff number is the count of circRNA that show different expressions between two samples. Sign. Diff number is the count of circRNA that show significantly different expressions between two samples, where FDR < =0.001 and |log2Ratio | > =1

## Comparisons with other databases

We compare horizontally with other circRNA databases (such as circBase (1), CSCD (32), CircNET (34) listed in Table 4). CCRDB can achieve the following functions: (i) discover new circRNA by sequencing the normal and pathological cells of the same person’s same tissues to avoid background effects of genetic differences among different people, (ii) provide a platform for circRNA differential analysis application and (iii) link and extend with external data sources, such as circBase, GO, pubmed, etc., to display a comprehensive network of RNA discovery and regulation. In general, the CCRDB provides users with interactive tools, a concise home page interface and a search engine to achieve a convenient and flexible query through sequence, gene and genome location. Taken together, the CCRDB can be an integrated resource for circRNA to provide not only valuable relationship between circRNAs and diseases, but also the new analysis tool to mine much more knowledge from the data as well.

**Figure 3:**
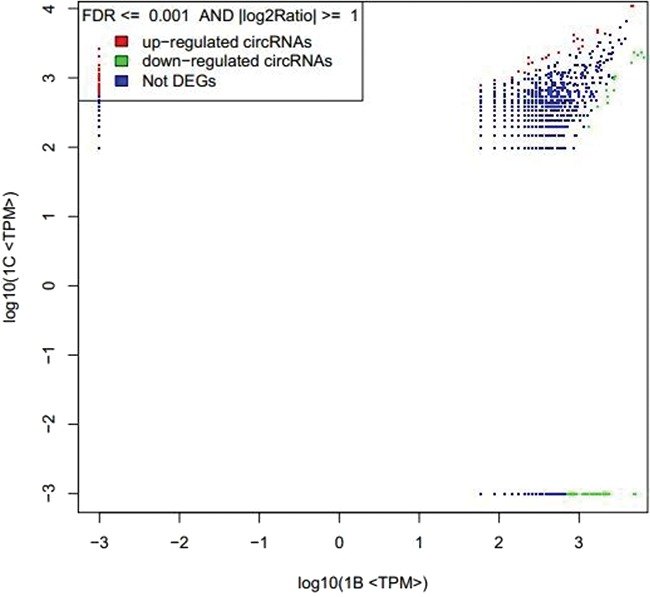
circRNAs expression level of 1B and 1C. The abscissa represents the signal expression of the control sample 1B, and the ordinate represents the expression of the treated sample 1C. Each point in the graph represents a circRNAs, and the red and green dots represent the significant expression circRNAs. The red dot indicates that the expression of circRNAs is up-regulated (compared with the control samples), the green dot indicates that the expression of circRNAs is down-regulated (compared with the control samples) and the blue dot indicates that there is no significant difference between the circRNAs.

**Figure 4:**
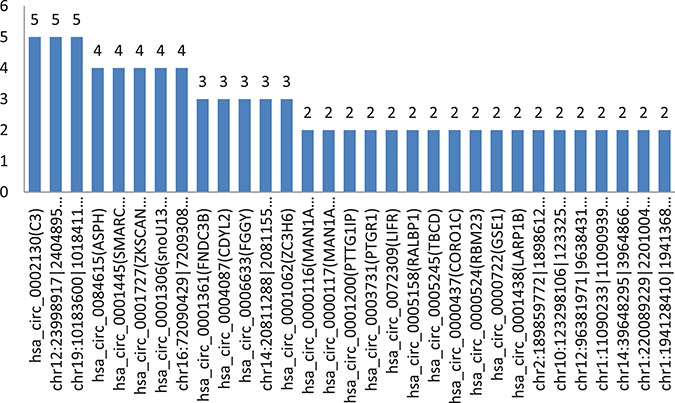
Shows the count of the comparison groups in which their circRNAs have common significant differences and the same regulation directions in all comparison groups of experimental samples.

**Figure. 5:**
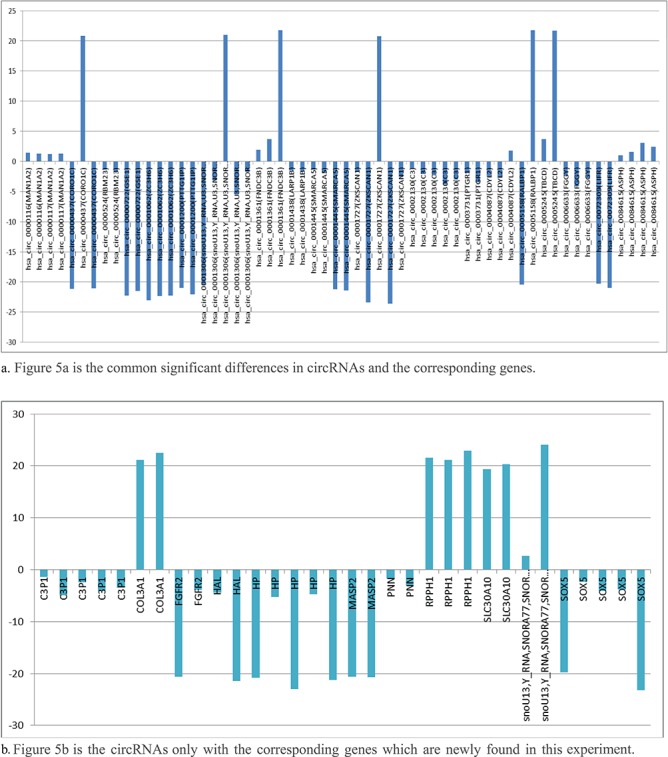
The common significant differences in circRNAs and their corresponding genes. (**a**) Figure 5a is the common significant differences in circRNAs and the corresponding genes. (**b**) Figure 5b is the circRNAs only with the corresponding genes that are newly found in this experiment.

**Table 6:** Discovery of common regulation direction in significant difference

CircRNA_ID	CircBase_ID	Gene ID	Up/down regulation	Found the comparison groups (x/y)
Chr19:6702138|6702590	hsa_circ_0002130	C3	Down	5/5
Chr8:62593527|62596747	hsa_circ_0084615	ASPH	Up	4/5
Chr4:144464662|144465125	hsa_circ_0001445	SMARCA5	Down	4/5
Chr7:99621042|99621930	hsa_circ_0001727	ZKSCAN1	Down	4/5
Chr3:171830242|171851336	hsa_circ_0001361	FNDC3B	Up	3/5
Chr12:23998917|24048958		SOX5	Down	5/5
Chr16:72090429|72093087		HP	Down	4/5

## Results

After the establishment of the new database, we further studied the circRNAs and the relationship between circRNAs and HCC and found some interesting results.

## Analysis method

We set up comparison groups for analysis. Two samples of sequencing circRNAs are used to form a comparison group. They can be from the same person (organ), or they can be chosen from different person’s (organ’s) sample. A comparison group selection method is that circRNAs are obtained from the same person’s circRNAs sequencing data to avoid background effects such as genetic differences among people. By using the circRNAs comparative analysis application, we compare the results between the circRNAs of the human cancer cells and the circRNAs of the same human’s adjacent normal cells.

Semaphore of the comparison group must be chosen for the comparative signal strength. The main principle of the circRNAs comparative analysis application is to compare the signal expression of the samples, which is the number of junction reads that supports circRNAs’ head to tail connections. It is the field name of ‘#junction_reads’ in the circRNAs information listed in Table **3**.

The *P*-value method is calculated in hypothesis test.The formula of *P*-value is shown below, where x and y are expressions of the two samples’ circRNAs in the comparison group, N_1_ and N_2_ are the summary expressions of the samples’ circRNAs in the comparison group.

\begin{equation*} p(y \vert x)=\left(\frac{N_{2}}{N_{1}}\right)^{y}\frac{(x + y)!}{x!y!\left(1+\frac{N_{2}}{N_{1}}\right)^{(x + y + 1)}}\end{equation*}

There are two major parameters, FDR and |log2Ratio|. log2Ratio| is the ratio of the semaphores when two samples are compared with log2. FDR is the false discovery rate of *P*-value. Usually |log2Ratio| is set to be greater than or equal to 1, and FDR is less than 0.001. These two parameters can be set according to actual needs.

### HCC cells shows distinctly different circRNAs from normal cells

Using the comparative analysis application, we select the same person (organ) as the comparison group samples, of which sample B was normal cells and sample C showed hepatoma cells. (We can also choose comparison groups in other ways). We labeled them 1B&1C, 2B&2C, … 5B&5C, respectively. The circRNAs expressed in the same organ (liver) of several groups of people were identified. The numbers of differences found in circRNAs between samples B and C were 6808, 4652, 4365, 3102 and 3534, respectively, compared with five different comparison groups. The numbers of significant differences were 111, 44, 21, 47 and 25, respectively. These differences and significant differences are analyzed, as shown in Table 5.

By setting the FDR and |log2Ratio| parameters, the results of the analysis with significant differences are obtained. The result of expression level 1B vs 1C is shown in Figure 3.

We put all comparison groups together. The significant differences of the same category in all groups are compared. And the numbers of comparison groups are analyzed where their differences are in the same regulatory direction.

All the significant differences between cancer cells and their adjacent normal cells of the same person were analyzed. Figure 4 shows the count of the comparison groups in which their circRNAs have common significant differences and the same regulation directions in all selected comparison groups of the experimental samples.

In the comparison group of five persons, there were 31 circRNAs with two or more comparison groups, which their significant differences have the same regulatory directions, including 20 circRNAs with circBASE_ID data and 11 without circBASE_ID data, as they are newly found.

There are three circRNAs with significant differences in the same direction of regulation that have been found in five comparison groups (5/5, in 100%). There are five circRNAs with significant differences in the same direction of regulation that have been found in four comparison groups (4/5, in 80%). There are five circRNAs with significant differences in the same direction of regulation that have been found in four comparison groups (3/5, in 60%).

The changes of circRNAs from normal cells to diseased cells in different comparison groups were generally consistent with the same regulatory directions (UP or DOWN). This helps us to find the corresponding regulatory or target genes from the significant variation of circRNAs, as shown in Figure 5a and b.

### Highly probable carcinomatous circRNAs

The circRNAs with same significant differences and same regulation directions, which occurred many times (comparison groups count) in the comparison groups through our analysis application, seem to strongly related to the disease. Corresponding candidate regulatory genes or target genes can be found from the circRNAs, as shown in the Figure 5.

We have found that, Has_circ_0002130-related geneID C3 showed significant differences in five of five comparison groups (5/5), which is down-regulated in our experimental samples. According to the report of the papers, the gene C3, inhibiting cancer in HCC, was found to be the biomarker candidates for distinguishing early HCC from cirrhosis. Hsa_circ_0001445 (related gene SMARCA5, 4/5 found in the experiment), hsa_circ_0001727 (related gene ZKSCAN1, 4/5 found in the experiment), chr12:23998917| -24048958 (related gene SOX, 5/5 found in the experiment) and chr16:72090429|72093087 (related gene HP, 4/5 found in the experiment), were down-regulated, which was consistent with the results of related papers. Hsa_circ_0084615 (related gene ASPH, 4/5 found in experiment) and hsacirc0001361(related gene FNDC3B, 3/5 found in experiment), were up-regulated, which was consistent with the results of related papers. Details are shown in Table 6 below.

## Summary and future directions

We sequenced the circRNAs of hepatocytes and constructed a new database CCRDB. Using the new database CCRDB and its analyzing tools, we further studied circRNAs and the relationship between circRNAs and HCC. It is of great significance for researchers to further analyze the rules of circRNAs, to understand the causes of circRNAs in disease discovery and to search for target genes for therapeutic approaches. Researchers can easily add circRNA sequencing data from other organs to this database and use the comparative analysis tools to provide powerful analytical functions to facilitate the discovery of new knowledge.

The future direction for development is to mine more circRNAs data from literatures and experiment to compile a more comprehensive database and offer a variety of analytical functions, including verification of analysis results, and intelligent tools by artificial intelligence technology.

## References

[1] Glažar P., Papavasileiou P. and Rajewsky N. (2014) CircBase: a database for circular RNAs. *RNA*, 20, 1666–1670. 25234927 10.1261/rna.043687.113 PMC4201819

[2] Liu Y., He J., Li C.E. et al. (2010) Identification and confirmation of biomarkers using an integrated platform for quantitative analysis of glycoproteins and their glycosylations. *J. Proteome Res.*, 9, 798–805. 19961239 10.1021/pr900715p PMC2838716

[3] Zhu C., Song H., Xu F. et al. (2018) Hepatitis B virus inhibits the expression of complement C3 and C4, in vitro and in vivo. *Oncol. Lett.*, 15, 7459–7463. 29731897 10.3892/ol.2018.8223 PMC5920570

[4] Zou Q., Hou Y., Wang H. et al. (2018) Hydroxylase activity of ASPH promotes hepatocellular carcinoma metastasis through epithelial-to-mesenchymal transition pathway. *EBioMedicine*, 31, 287–298. 29764768 10.1016/j.ebiom.2018.05.004 PMC6013968

[5] Yu J., Xu Q.G., Wang Z.G. et al. (2018) Circular RNA cSMARCA5 inhibits growth and metastasis in hepatocellular carcinoma. *J. Hepatol.*, 68, 1214–1227. 29378234 10.1016/j.jhep.2018.01.012

[6] Yao Z., Luo J., Hu K. et al. (2017) ZKSCAN1 gene and its related circular RNA (circZKSCAN1) both inhibit hepatocellular carcinoma cell growth, migration, and invasion but through different signaling pathways. *Mol. Oncol.*, 11, 422–437. 28211215 10.1002/1878-0261.12045 PMC5527481

[7] Lin C.H., Lin Y.W., Chen Y.C. et al. (2016) FNDC3B promotes cell migration and tumor metastasis in hepatocellular carcinoma. *Oncotarget*, 7, 49498–49508. 27385217 10.18632/oncotarget.10374 PMC5226524

[8] Wang D., Han S., Wang X. et al. (2015) SOX5 promotes epithelial-mesenchymal transition and cell invasion via regulation of Twist1 in hepatocellular carcinoma. *Med. Oncol.*, 32, 461. 25572815 10.1007/s12032-014-0461-2

[9] Tai C.S., Lin Y.R., Teng T.H. et al. (2017) Haptoglobin expression correlates with tumor differentiation and five-year overall survival rate in hepatocellular carcinoma. *PLoS One*, 12, e0171269. 28158312 10.1371/journal.pone.0171269 PMC5291462

[10] Memczak S., Jens M., Elefsinioti A. et al. (2013) Circular RNAs are a large class of animal RNAs with regulatory potency. *Nature*, 495, 333–338. 23446348 10.1038/nature11928

[11] Hansen T.B., Jensen T.I., Clausen B.H. et al. (2013) Natural RNA circles function as efficient microRNA sponges. *Nature*, 495, 384–388. 23446346 10.1038/nature11993

[12] Liu Q., Zhang X., Hu X. et al. (2016) Circular RNA related to the chondrocyte ECM regulates MMP13 expression by functioning as a MiR-136 ‘sponge’ in human cartilage degradation. *Sci. Rep.*, 6, 22572. 26931159 10.1038/srep22572 PMC4773870

[13] Hansen T.B., Kjems J. and Damgaard C.K. (2013) Circular RNA and miR-7 in cancer. *Cancer Res.*, 73, 5609–5612. 24014594 10.1158/0008-5472.CAN-13-1568

[14] Bachmayr-Heyda A., Reiner A.T., Auer K. et al. (2015) Correlation of circular RNA abundance with proliferation—exemplified with colorectal and ovarian cancer, idiopathic lung fibrosis, and normal human tissues. *Sci. Rep.*, 5, 8057. 25624062 10.1038/srep08057 PMC4306919

[15] Guarnerio J., Bezzi M., Jeong J.C. et al. (2016) Oncogenic role of fusion-circRNAs derived from cancer-associated chromosomal translocations. *Cell*, 165, 289–302. 27040497 10.1016/j.cell.2016.03.020

[16] Jeck W.R. and Sharpless N.E. (2014) Detecting and characterizing circular RNAs. *Nat. Biotechnol.* 32, 453–461. 24811520 10.1038/nbt.2890 PMC4121655

[17] Pineau P. and Tiollais P. (2010) Hepatitis B vaccination: a major player in the control of primary liver cancer. *Pathol. Biol.*, 58, 444–453. 19896296 10.1016/j.patbio.2009.03.004

[18] Bahn J.H., Zhang Q., Li F. et al. (2015) The landscape of microRNA, Piwi-interacting RNA, and Circular RNA in Human Saliva. Clin Chem, 61, 221–230. 25376581 10.1373/clinchem.2014.230433 PMC4332885

[19] Li Y., Zheng Q., Bao C. et al. (2015) Circular RNA is enriched and stable in exosomes: a promising biomarker for cancer diagnosis. *Cell Res.*, 25, 981–984. 26138677 10.1038/cr.2015.82 PMC4528056

[20] Zhang X., Hu S.J., Zhang X. et al. (2014) MicroRNA-7 arrests cell cycle in G1 phase by directly targeting CCNE1 in human hepatocellular carcinoma cells. *Biochem. Biophys. Res. Commun.*, 443, 1078–1084. 24370822 10.1016/j.bbrc.2013.12.095

[21] Qin M., Liu G., Huo X. et al. (2018) Hsa_circ_0001649: a circular RNA and potential novel biomarker for hepatocellular carcinoma. *Biochem. Biophys. Res. Commun.*, 497, 122–126. 29421663 10.1016/j.bbrc.2018.02.036

[22] Li Y., Dong Y.C.H., Huang Z.Y. et al. (2017) Computational identifying and characterizing circular RNAs and their associated genes in hepatocellular carcinoma. PLoS One, 12, e0174436. 28346469 10.1371/journal.pone.0174436 PMC5367815

[23] Han D., Li J.X., Wang H.M. et al. (2017) Circular RNA circMTO1 acts as the sponge of microRNA-9 to suppress hepatocellular carcinoma progression. *Hepatology*, 66, 1151–1164. 28520103 10.1002/hep.29270

[24] Huang X.Y., Huang Z.L., Xu Y.H. et al. (2017) Comprehensive circular RNA profiling reveals the regulatory role of the circRNA-100338/miR-141-3p pathway in hepatitis B-related hepatocellular carcinoma. *Nat. Sci. Rep.*, 7, 5428. 10.1038/s41598-017-05432-8 PMC5511135 28710406

[25] Fu L.Y., Wu S.D., Yao T. et al. (2017) Decreased expression of hsa_circ_0003570 in hepatocellular carcinoma and its clinical significance. *J Clin Lab Anal.*, e22239. 10.1002/jcla.22239 PMC6816847 28493512

[26] Fu L.Y., Chen Q.Q., Yao T. et al. (2017) Hsa_circ_0005986 inhibits carcinogenesis by acting as a miR-129-5p sponge and is used as a novel biomarker for hepatocellular carcinoma. *Oncotarget*, 8, 43878–43888. 28410211 10.18632/oncotarget.16709 PMC5546447

[27] Lu J., Xia Y., Chen K. et al. (2016) Oncogenic role of the Notch pathway in primary liver cancer. *Oncol. Lett.*, 12, 3–10. 27347091 10.3892/ol.2016.4609 PMC4906927

[28] Jia M., Jiang L., Wang Y.D. et al. (2016) lincRNA-p21 inhibits invasion and metastasis of hepatocellular carcinoma through Notch signaling-induced epithelial-mesenchymal transition. *Hepatol. Res.*, 46, 1137–1144. 27391793 10.1111/hepr.12659

[29] Ghosal S., Das S., Sen R. et al. (2013) Circ2Traits: a comprehensive database for circular RNA potentially associated with disease and traits. *Front. Genet.*, 4, 283. 24339831 10.3389/fgene.2013.00283 PMC3857533

[30] Li J.H., Liu S., Zhou H. et al. (2014) StarBase v2.0: decoding miRNA-ceRNA, miRNA-ncRNA and protein-RNA interaction networks from large-scale CLIP-Seq data. *Nucleic Acids Res.*, 42, D92–D97. 24297251 10.1093/nar/gkt1248 PMC3964941

[31] Chen X.P., Han P., Zhou T. et al. (2016) circRNADb: a comprehensive database for human circular RNAs with protein-coding annotations. *Sci. Rep.*, 6, 34985. 27725737 10.1038/srep34985 PMC5057092

[32] Xia S.Y., Feng J., Chen K. et al. (2018) CSCD: a database for cancer-specific circular RNAs. *Nucleic Acids Res.*, 46, 925–929. 10.1093/nar/gkx863 PMC5753219 29036403

[33] Liu Y.C., Li J.R., Sun C.H. et al. (2016) CircNet: a database of circular RNAs derived from transcriptome sequencing data. *Nucleic Acids Res*, 44, 209–215. 10.1093/nar/gkv940 PMC4702939 26450965

[34] Salzman J., Gawad C., Wang P.L. et al. (2012) Circular RNAs are the predominant transcript isoform from hundreds of human genes in diverse cell types. *PLoS One*, 7, e30733. 22319583 10.1371/journal.pone.0030733 PMC3270023

[35] Jeck W.R., Sorrentino J.A., Wang K. et al. (2013) Circular RNAs are abundant, conserved, and associated with ALU repeats. *RNA*, 19, 141–157. 23249747 10.1261/rna.035667.112 PMC3543092

[36] Maass P.G., Glažar P., Memczak S. et al. (2017) A map of human circular RNAs in clinically relevant tissues. *J. Mol. Med. (Berl).*, 95, 1179–1189. 28842720 10.1007/s00109-017-1582-9 PMC5660143

[37] Rybak-Wolf A., Stottmeister C., Glažar P. et al. (2015) Circular RNAs in the mammalian brain are highly abundant, conserved, and dynamically expressed. *Mol. Cell*, 58, 870–885. 25921068 10.1016/j.molcel.2015.03.027

[38] Zhang Y., Zhang X.O., Chen T. et al. (2013) Circular intronic long noncoding RNAs. *Mol. Cell*, 51, 792–806. 24035497 10.1016/j.molcel.2013.08.017

[39] Jeck W.R., Sorrentino J.A., Wang K. et al. (2013) Circular RNAs are abundant, conserved, and associated with ALU repeats. *RNA*, 19, 141–157. 23249747 10.1261/rna.035667.112 PMC3543092

[40] Salzman J., Chen R.E., Olsen M.N. et al. (2013) Cell-type specific features of circular RNA expression. *PLoS Genet.*, 9, e1003777. 24039610 10.1371/journal.pgen.1003777 PMC3764148

[41] Memczak S., Jens M., Elefsinioti A. et al. (2013) Circular RNAs are a large class of animal RNAs with regulatory potency. Nature, 495, 333–338. 23446348 10.1038/nature11928

